# Seroprevalence and Risk Factors of *Toxoplasma gondii* in Ruminant Meats from Wet Markets in Klang Valley and Abattoirs in Selangor, Malaysia

**DOI:** 10.3390/ani10071139

**Published:** 2020-07-06

**Authors:** Norhamizah Abdul Hamid, Mohammed Babatunde Sadiq, Siti Zubaidah Ramanoon, Rozaihan Mansor, Malaika Watanabe, Nur Mahiza Md Isa, Juriah Kamaludeen, Sharifah Salmah Syed-Hussain

**Affiliations:** 1 Department of Veterinary Clinical Studies, Faculty of Veterinary Medicine, Universiti Putra Malaysia, Serdang 43400 UPM, Selangor, Malaysia; norhamizah79@gmail.com; 2 Department of Farm and Exotic Animal Medicine and Surgery, Faculty of Veterinary Medicine, Universiti Putra Malaysia, Serdang 43400 UPM, Selangor, Malaysia; sadiquemohammed99@yahoo.com (M.B.S.); sramanoon@upm.edu.my (S.Z.R.); rozaihan@upm.edu.my (R.M.); 3 Department of Companion Animal Medicine and Surgery, Faculty of Veterinary Medicine, Universiti Putra Malaysia, Serdang 43400 UPM, Selangor, Malaysia; maraika@upm.edu.my; 4 Department of Veterinary Pathology and Microbiology, Faculty of Veterinary Medicine, Universiti Putra Malaysia, Serdang 43400 UPM, Selangor, Malaysia; nurmahiza@upm.edu.my; 5 Department of Animal Science and Fisheries, Faculty of Agriculture and Food Science, Universiti Putra Malaysia Bintulu, Sarawak Campus, Bintulu 97008, Sarawak, Malaysia; juriahk@upm.edu.my

**Keywords:** *Toxoplasma gondii*, ruminants, wet markets, abattoir, meat juice, ELISA

## Abstract

**Simple Summary:**

This study investigated the prevalence of *Toxoplasma. gondii* in meats of cattle, goat and sheep from wet markets and abattoirs in Selangor, Malaysia. Meat samples from wet markets in various districts and diaphragm samples from abattoirs were analyzed using ELISA to check for *T. gondii* IgG antibodies. Furthermore, attempts were made to detect *T. gondii* DNA from meat samples using the nested PCR technique. Twenty-five percent of the samples were positive for *T. gondii* antibodies, with the highest recorded in goat (55%), followed by sheep (35%) and cattle meat (9%). *T. gondii* DNA was not detected in any of the meat samples. Being the first report in Malaysia, the findings highlight the need for proper control in reducing exposure of ruminant meats to the parasite, especially those destined for human consumption.

**Abstract:**

(1) Background: The objective of this study was to determine the prevalence of *T. gondii* in meats of cattle, goat and sheep from wet markets in Klang Valley, and abattoirs in Selangor, Malaysia; (2) Methods: A total of 192 meat samples were purchased from 51 wet markets in six districts in Klang Valley (Gombak, Klang, Kuala Lumpur, Hulu Langat, Petaling and Putrajaya). Meanwhile, a total of 200 diaphragm samples were collected from two government abattoirs located in Shah Alam and Banting, Selangor. All meat juices from samples were subjected to an indirect-ELISA kit for the presence of *T. gondii* IgG antibodies. Furthermore, all 184 meat samples of goat and sheep were subjected to conventional nested PCR (B1 genes) for the detection of *T. gondii* DNA; (3) Results: *T. gondii* antibodies were detected in 25% (n = 98/392) of the samples with seroprevalence of 9.1% (19/208, CI: 5.9%–13.8%) in cattle meat; 54.7% (41/75, 95% CI: 43.5%–65.4%) in goat meat and 34.9% (38/109, CI: 26.6%–44.2%) in sheep meat. No *T. gondii* DNA was detected in any of the meat samples of goat and sheep. *T. gondii* seropositivity in wet market samples was higher in goat (OR = 37.1 CI 12.4–110.3) and sheep meat (OR 9.03 CI: 3.28–24.8) compared to cattle meat (OR = 1.0) At univariate level, meat from non-licensed abattoirs (OR = 6.0 CI: 2.9–12.3) and female animals (OR = 6.7; CI 1.9–22.6) had higher risks of being seropositive for *T. gondii* antibodies than licensed abattoirs and male animals, respectively. (4) Conclusions: This is the first report of seroprevalence of *T. gondii* in ruminant meats for human consumption in Malaysia. The findings signified high exposure of meat samples from wet markets to *T. gondii* and the need for control measures to reduce the likelihood of infection when such raw or undercooked meats are consumed.

## 1. Introduction

*Toxoplasma gondii* is an apicomplexan protozoan capable of infecting all warm-blooded animals and causing major health concerns to humans, especially to unborn fetuses and immunocompromised individuals. In humans, toxoplasmosis has been shown to cause fever, lymphadenopathy, headache, myalgia, arthralgia, dizziness, and in worst situations, encephalitis, blindness and abortions [1]. Consumption of raw, or undercooked meat or meat products is highly associated with toxoplasmosis in humans. In European countries, it has been estimated that 30–60% of the infection in humans were due to consumption of undercooked meat or meat products [1,2]. Transmission in human may also occur from infected mother to the unborn fetus [3]. A study conducted in the United States had shown that the cost of testing and treating a newborn child due to toxoplasmosis ranges from USD 300,000 to more than USD 3 million for developmental disorder leading to hearing, vision or cognitive losses [4]. In South Australia, it was estimated that toxoplasmosis costs the sheep industry up to AUD 70 million per year [5]. These data indicate that *T. gondii* causes significant financial loss to animal producers, as well as to the public.

Ruminant animals especially sheep and goats are highly susceptible to *T. gondii* infection leading to reproductive failures [6]. There are indications of increasing seroprevalence of *T. gondii* worldwide, as the estimates of 18.6%, 43.9% and 47% were observed in cattle, goats and sheep, respectively [6,7,8]. Ruminant livestock are more likely to be infected due to the nature of grazing in contaminated environment with the oocysts [9]. Thus, the high rate of *T. gondii* infection reported in small ruminants worldwide, not only affects the economics of ruminant production but also imposes significant zoonotic health hazard in humans consuming infected meat.

In Malaysia, *T. gondii* seroprevalence among healthy Malaysians ranged from 14% to 30% [10,11], indicating that the disease is highly prevalent. Furthermore, alarming results have indicated that the seroprevalence of *T. gondii* among pregnant women in South East Asia was highest in Malaysia (42.5%) [12] as compared to the other neighboring countries such as Thailand (28.3%0 [13] and the Philippines (23.8%) [14]. Since *T. gondii* remains an important zoonotic pathogen worldwide, it is imperative to evaluate the exposure of ruminants to the parasite as well as in meats destined for human consumption in Malaysia. In Malaysia, most works on *T. gondii* in meat have been conducted in poultry, wild boar and exotic animals [15,16,17] but none in ruminants. Therefore, the aim of the present work was to determine the prevalence of *T. gondii* in meats of cattle, goat and sheep from wet markets and abattoirs in Klang Valley and Selangor, Malaysia and the risk factors involved.

## 2. Materials and Methods 

### 2.1. Study Design, Study Area and Selection of Sample Sites

**Figure 1:**
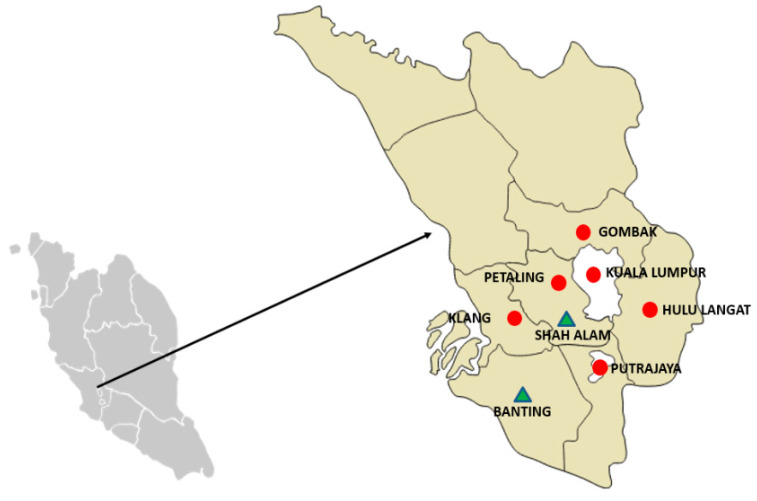
The map of Peninsular Malaysia (left), and the map of the state of Selangor (right). Red circles denote the sampling areas (wet markets) in six districts in Klang Valley, while green triangles denote the sampling area (abattoirs) in Selangor, Malaysia.

A cross sectional study was conducted involving the selection of wet markets in Klang Valley and abattoirs in Selangor, Malaysia. The sampling sites were selected based on the availability of fresh meat from animals slaughtered locally in the study area. As shown in Figure 1, Klang Valley consists of four districts within Selangor State territory (Gombak: 3.2535° N, 101.6533° E, Hulu Langat: 2.9936° N, 101.7892° E, Klang: 3.0449° N, 101.4456° E, and Petaling: 3.1846° N, 101.5360° E) and two federal territories (Kuala Lumpur:3.1390° N, 101.6869° E and Putrajaya: 2.9264° N, 101.6964° E). 

In Klang Valley, there were three types of wet markets available: (a) “*pasar besar*”—market located within a building, (b) “*pasar tani*”—open stalls which operate in the morning, and (c) “*pasar malam*”—open stalls which operate at night. “*Pasar malam*” were not included in the sampling, as they only cater for small areas within the districts, and usually locally slaughtered meats would not be available. For the wet markets, only fresh meat samples were collected in the present work. Fresh meat is also locally known as “warm meat”, as opposed to frozen meat. When locally slaughtered meats were not available at markets within the districts, repeated samplings were conducted on the same stall within a two-week interval. Information regarding the meat samples was obtained through informal conversation with the sellers/butchers. The fresh meats were categorized into two groups: with a “Lagenda” tag, and without, based on observation. “Lagenda” tag on the meat indicates that the animal has been approved by the Department of Veterinary Services (DVS), has a Veterinary Health Certificate and Slaughter Permit, and slaughtered at a licensed abattoir. Two licensed abattoirs, (located in Banting and Shah Alam, Selangor) were selected in the present work, since they cater for major ruminant slaughters in Selangor. Animals slaughtered in Banting abattoir were reared locally in Selangor, while those in Shah Alam were imported.

The required sample size was calculated using the EpiTools website (http://epitools.ausvet.com.au) while assuming an expected seroprevalence of 35.5% from a previous study conducted in Malaysia by Chandrawathani et al. [18] at confidence level (CI) of 95%, precision level of 5%, and a target population of ruminants (cattle, goat and sheep) in Selangor as 40,000 animals [19]. The calculated sample size was 350, and they were equally divided into samples from wet markets and abattoirs. 

### 2.2. Meat Sampling

From the wet markets, only meat samples from locally slaughtered animals were purchased. Sirloin meats were selected whenever available and ground meats were not included to prevent mixture of meat from various animal sources. Samples were stored in individual sealed plastic bags and frozen at −20 °C until further analysis. Information on species, source of meat (obtained from licensed or non-licensed abattoir), location of market and stall numbers were recorded. From the abattoirs, diaphragm samples were collected during individual animal slaughtering, stored in individual sealed plastic bags, and frozen at −20 °C until further analysis. Information on the animals such as species and sex were also recorded.

### 2.3. Serology

All meat and diaphragm samples collected were thawed overnight to collect the meat juice which was then aliquoted into individual micro-centrifuge tubes. These samples were tested using a commercial ELISA test kit (ID Screen^®^ Toxoplasmosis Indirect Multispecies Test Kit, France). This indirect assay uses P30 antigen of *T. gondii* with anti-multi-species immunoglobulin G (IgG) conjugates that detect antibodies against *T. gondii* in samples from multiple species including ruminants, cats, dogs and swine. Results were calculated based on a sample-to-positive ratio (S/P), where the S/P percentage (OD sample/OD positive control × 100) was calculated for each sample. Based on the manufacturer’s instructions, samples with S/P ≥ 50% were considered positive, S/P 40 to <49% were considered doubtful, and S/P < 40% were considered negative. In the present work, doubtful results were recorded as negative for *T. gondii* antibodies.

### 2.4. Detection of T. gondii DNA in Meat Samples

All meat and diaphragm samples from goat and sheep samples were subjected to nested PCR designed to amplify the B1 gene of *T. gondii* as described by Jones et al. [20]. The meat and diaphragm from cattle were not subjected to PCR due to the suggested ability of cattle to eliminate *T. gondii* [3].

#### 2.4.1. DNA Extraction

Approximately 10 g of each meat and diaphragm samples from different parts were finely minced in a Petri dish using a sterile single use scalpel blade. Any connective or fat tissue present was removed. Samples were then processed using the DNeasy Blood and Tissue Kit (Qiagen, Germany) as per manufacture’s instruction. Extracted DNA from meat samples were stored at −20 °C until further analysis.

#### 2.4.2. Polymerase Chain Reaction

The extracted DNA was subjected to a modified conventional nested PCR for the detection of *T. gondii* DNA. The PCR was designed to amplify the B1 gene (Burg et al., 1989) of *T. gondii* using the primer sequence as described by Jones et al (2000), and shown in Table 1. DNA extracted from a New Zealand *T. gondii* isolate was used as the positive control. The DNA was added to the PCR assay, and two negative controls from the first-round amplification were also included in the nested reaction. PCR products were then run on a 3.0 % (*w*/*v*) agarose gel (Hydragene, USA) containing SYBR Safe DNA gel stain (Invitrogen, USA) at 100 V for 30 min, and visualized on a trans-illuminator (Biorad, California, US). To confirm that there was no contamination occurring during laboratory testing, no template control (NTC) was also included as processing controls.

### 2.5. Statistical Analysis

The obtained data were analyzed using SPSS Version 25 (IBM, USA). Descriptive statistics were used to summarize the data and to determine the seroprevalence of *T. gondii* in the samples collected from wet markets and abattoirs. Separate binary logistic regression models were conducted to determine the association between the potential risk factors and seroprevalence of *T. gondii* in wet market and abattoir samples. At multivariate level, *p* < 0.05 was considered for any significant relationship, while odds ratio (OR) and 95% confidence interval (CI: 95%) were used to express the strength of the association.

## 3. Results

### 3.1. Descriptive Results

A total of 392 samples (meat, n = 192; diaphragm, n = 200) were collected consisting of cattle, goat and sheep meats from wet markets in Klang Valley, and two main licensed abattoirs in Selangor, as summarized in Table 2. For wet market samples, 51 wet markets (62 stalls selling meat) were visited, but only 42 wet markets (55 stalls) were selling fresh locally slaughtered meat; thus, giving a total of 192 samples. From 192 meat samples, 118 and 74 were with and without “Lagenda” tag, respectively. Majority of the wet market (56%; 108/192) and abattoir (50%; 100/200) meat samples were from cattle, while 61% (118/192) of the former were from licensed abattoirs (Table 2). A higher proportion of the meat samples from the abattoirs were from female (73.5%; 147/200) as compared to male (26.5%; 53/200) animals.

### 3.2. Seroprevalence of T. gondii in Wet Markets and Abattoirs Meat Samples

The seropositive samples in cattle, goat and sheep purchased from wet markets in Klang Valley, and abattoirs in Selangor are presented in Table 3. *T. gondii* antibodies was detected in 25% of the meat samples giving an overall seroprevalence of 9.1% (19/208, CI: 5.6–13.9%) in cattle; 54.7% (41/75, CI: 42.7–66.2%) in goats, and 34.9% (38/109, CI: 26–44.6%) in sheep. Seroprevalence of *T. gondii* from the wet market samples were 6% (6/108, CI: 2.1–11.7%), 69% (24/35, CI: 50.7–83.1%) and 35% (17/49, CI: 21.7–49.6%) in meats of cattle, goat and sheep, respectively, giving an overall seroprevalence of 24.5%. The highest seroprevalence was detected in Klang district at 44% (11/26, CI: 24.4-65.1) followed by Hulu Langat (14/42, CI: 19.6–49.5), and the lowest was in Gombak (7.7%; 1/13, CI: 0.2–36), followed by Kuala Lumpur at 8.3% (2 out of 24, CI: 1.0–27.0). Results from the abattoir samples revealed an overall seroprevalence of *T. gondii* at 31.6%, with individual results of 13% (13/100, CI: 11.8%–28.1%), 43% (17/40, CI: 27.0–59.1) and 35% (21/60, CI: 22.9–45.2) in meats of cattle, goat and sheep, respectively.

### 3.3. Risk Factors Associated with T. gondii Seropositivity

As shown in Table 4, results from the wet markets indicated that there was at least one seropositive sample from every district. Repeated sampling from markets in Kajang and Petaling districts revealed that seropositive goat meat samples were consistently found (data not shown). However, no significant associations were found between districts and the number of seropositive *T. gondii* meat samples detected. Meanwhile, the seroprevalence of samples obtained from the abattoirs were 16% (8/50, CI: 7.2–29.1%) and 36% (55/150, CI: 29.0–44.9%) for Banting and Shah Alam, respectively (Table 5). No association was detected between the location of abattoirs and *T. gondii* seropositive meat samples.

For the wet market samples, there was a significant association between *T. gondii* seropositivity and species. At univariate level, sheep (OR = 37.0; CI 12.4–110.2) and goat (OR = 9.0; CI 3.2–24.8) meat samples had higher odds of being seropositive for *T. gondii* compared to cattle meat (OR = 1.0). Multivariate analysis of samples from abattoirs indicated that meat from goat and sheep were five and four times, respectively more likely to be seropositive for *T. gondii* than cattle meat. At univariate level, results from the wet markets for the meat source indicated higher odds of meat from non-licensed abattoirs (OR = 6.0 CI: 2.9–12.3) and female animals (OR = 6.7; CI 1.9–22.6) being seropositive for *T. gondii* antibodies than licensed abattoirs and male animals, respectively. 

### 3.4. Detection of T. gondii DNA in Goat and Meat Samples

The DNA of *T. gondii* was not detected in any of the 184 goat and sheep meat samples purchased from wet markets and abattoirs. 

## 4. Discussion

The present work aimed to determine the prevalence of *T. gondii* in meats of cattle, goat and sheep from wet markets and abattoirs in Klang Valley and Selangor, Malaysia, and the risk factors involved. To the best of our knowledge, this is the first attempt to determine the prevalence of *T. gondii* in ruminant meats in Malaysia, specifically in Selangor. 

Wet markets and supermarkets are outlets that play important roles as direct suppliers of meats to consumers. Hence, the need to ensure that such meat is safe and wholesome for consumption. The overall seroprevalence from the wet market samples analyzed in the present work was 24.5%, with higher values in goats (69%) and sheep (35%) as compared to cattle (6%). A similar study conducted on goat’s meat in retail stores in the USA reported a seroprevalence of 53% [21], whereas prevalence of tissue cysts in retail sheep meat in Turkey was 21% [22,23]. These findings are consistent with the results obtained in the present work, suggesting a possible risk of *T. gondii* transmission to humans. 

The current prevalence of *T. gondii* antibodies found in meats of goats (42.5%) and sheep (35%) from the two abattoirs is similar to that reported in Iran (goats; 48%, sheep; 32.6%) [24] and Pakistan (goats; 42.8%, sheep; 26.2%) [25]. However, the prevalence estimate in the present work is higher than that reported in goats in Myanmar (11.4%) [26], and lower than the estimates in Egypt (62%) [8] and Italy (63.3%) [27]. Such variations could be due to the presence of potential risk factors for exposure to the parasites, types of serological assay and cut-off used, sample size, climate variation, and farm levels of contamination with *T. gondii* oocysts especially in soil, feed and water trough [28,29].

The overall seroprevalences found in meat samples analyzed in the present work are higher than those previously reported in farms in Malaysia of 55%, 35% and 9% in goats, sheep, and cattle, respectively [18,30,31]. This suggests an increased exposure of ruminants to *T. gondii* which could be related to increased contamination of the environment by the parasites. Furthermore, the meat samples analyzed in the present work were presumably from adult animals; hence, they were more exposed to the parasites compared to those sampled in the farms [32,33,34]. Studies have shown that age is a risk factor for ovine toxoplasmosis [35,36].

In the present work, indirect ELISA assay was used to detect the presence of *T. gondii* antibodies in the meat juice from meat samples. The use of ELISA has been widely documented in epidemiological studies for the detection *T. gondii* antibodies in ruminants [7,8,37,38]. Similarly, the use of meat juice has been reported in various studies involving cattle, goats, sheep, pigs, wild boars, and exotic animals [39,40]. In the present work, the use of meat juice has proven to be convenient and most appropriate especially when dealing with slaughtered animals and testing of meats sold for human consumption. 

Various assays and protocols have been applied for the molecular diagnosis of *T. gondii* by targeting specific DNA sequence of the pathogen with highly conserved regions. This includes the B1 gene, 529 bp repetitive element and 18S rDNA gene sequences [41]. Nested PCR targeting on the B1 gene has been used in various meat studies showing high sensitivity in detecting *T. gondii* DNA ruminants [42,43]. Similar studies in sheep and cattle revealed 33% to 60% and 16% to 37% sensitivity, respectively, for the detection of *T. gondii* DNA in the meat samples [35,44,45]. In the present work, however, no *T. gondii* DNA was detected in any of the meat samples from goats and sheep. Similar results have been reported in other related studies [46]. Favorable sites for *T. gondii* tissue cysts containing bradyzoites were commonly reported in the brain, liver, heart, diaphragm and skeletal muscles, with naturally infected animals harboring low number of tissue cysts, which could be difficult to detect using direct techniques [3]. In this study, during wet market sampling, it was observed that organs or offal were not commonly available. Thus, only meat samples were selected. Majority of the selected meat samples were sirloin meats (skeletal muscles; Longissimus dorsi muscle) and these body parts have been used in several studies for the detection of *T. gondii* in meat samples [47,48]. As for the abattoirs, 100 diaphragm samples were negative for *T. gondii* DNA. The challenges in detecting *T. gondii* DNA in tissues have been reported in various studies with reasons such as inhomogeneous/uneven distribution of the *T. gondii* tissue cysts, as well as the relatively small sample size used for the DNA extraction [29,49]. 

In one study conducted using the same targeted B1 gene and mice bioassays, no *T. gondii* DNA was detected from 48 pork meat samples [46]. Bioassay in mice or cats is regarded as goal standard in the detection of viable *T. gondii*, but it is time consuming, laborious and costly, and thus not suitable for epidemiological studies [29].

Factors such as species of ruminants and meat sources were associated with *T. gondii* seropositivity in the wet market samples analyzed in the present work. Higher odds of seropositivity were recorded in goat and sheep meat samples compared to samples from cattle. This result is consistent with previous studies conducted worldwide reporting significantly higher seroprevalence in small ruminants as compared to cattle [8,32]. Small ruminants, especially sheep, are more susceptible to *T. gondii* and they suffer from abortion and neonatal losses, but such reports are lacking in cattle [50,51]. The feeding habit of small ruminants, which consume the lower parts of grasses or plants, has been suggested to increase the possibility of ingesting infective oocysts. Therefore, clinical disease occurs more in sheep and goats as compared to in cattle [3]. In addition, cattle have been considered as poor hosts for *T. gondii* as they have been shown to have the ability to eliminate or reduce the numbers of tissue cysts to undetectable level few weeks post-infection which is suggestive of innate resistance [3].

Sources of meat and locations are closely related factors that could promote *T. gondii* transmission to meat-producing animals [52]. In this study, the wet market meat samples obtained from non-licensed abattoirs had higher odds of being seropositive as compared to those from licensed abattoirs. Non-licensed or self-slaughter at the farm are farms that might not comply with the local veterinary authority, and that they do not possess the Veterinary Health Certificate and Slaughter Permit. These farms may lack proper farm management such as controlling movement of animals in the farm. Back yard farming with the presence of cats could contribute to the high infection rate of *T. gondii*. These findings gave an insight on the importance of sampling of meat ready for human consumption as compared to detection at the farm level only.

For the abattoir-based meat samples, the seroprevalence of *T. gondii* was associated with species and gender. A significant difference in *T. gondii* seropositivity was observed between the meat samples from two sex groups, with female more likely to be seropositive than male. This finding is in accordance to a recent report by Tilahun et al. [53] who found that female sheep had 2.6 times higher odds of being seropositive to *T. gondii* compared with male sheep in Ethiopia. Features such as periodic immunosuppression could increase the susceptibility of females to infection by the parasite [54]. In contrast, the higher seroprevalence observed in male sheep in another study was attributed to reduced immunity linked to androgen production [55]. These inconsistent results depict the need for further investigation on the susceptibility to *T. gondii* between male and female ruminants. Moreover, the uneven sample size distribution between female and male animals sampled at the abattoirs in the present work might have influenced the obtained findings.

## 5. Conclusions

This is the first study to report the seroprevalence of *T. gondii* in ruminant meats destined for human consumption in Malaysia. The seroprevalence rate of *T. gondii* from wet markets and abattoirs in Selangor was found to be high. Despite *T. gondii* DNA not being detected in the meats of goats and sheep, the result highlights the increasing exposure of slaughtered animals to the parasite and the likelihood of infection when such raw or undercooked meats are consumed.

## Figures and Tables

**Table 1:** Outer (forward and reverse) and inner (forward and reverse) primer sequences for Toxoplasma gondii B1 gene used for the detection of *T. gondii* DNA in meat samples.

Oligonucleotide Primer	Sequence	Sequence Position	PCR Product
Outer primer (forward)	5′-GGAACTGCATCCGTTCATGAG-3′	694–714	193 bp
Outer primer (reverse)	5′-TCTTTAAAGCGTTCGTGGTC-3′	887–868	
Inner primer (forward)	5′-TGCATAGGTTGCAGTCACTG-3′	757–776	96 bp
Inner primer (reverse)	5′-TGCATAGGTTGCAGTCACTG-3′	853–831	

**Table 2:** Description of meat samples purchased from wet markets in Klang Valley, and abattoirs in Selangor.

Wet Market Samples (Meat)			Abattoir Samples (Diaphragm)		
Category	Number of Samples (*n*)	Percentage (%)	Category	Number of Samples (*n*)	Percentage (%)
**Meat**			**Species**		
Beef	108	56.3	Cattle	100	50.0
Chevron	35	18.2	Goat	40	20.0
Mutton	49	25.5	Sheep	60	30.0
**Location**			**Location**		
Gombak	13	6.7	Banting (local)	50	25.0
Hulu Langat	42	21.8	Shah Alam (imported)	150	75.0
Klang	25	13.0			
Kuala Lumpur	24	12.5			
Petaling	79	41.4			
Putrajaya	9	4.7			
**Type of wet markets**			**Sex**		
*Pasar Besar*	151	78.6	Male	53	26.5
*Pasar Tani*	41	21.4	Female	147	73.5
**Meat source**					
Licensed abattoir	118	61.4			
Non-licensed abattoir	74	38.5			

**Table 3:** Seropositive samples in cattle, goat and sheep purchased from wet markets in Klang Valley, and abattoirs in Selangor.

Location	Beef	Chevon	Mutton	Total	Seropositive (%)
**Wet Market**					
District					
Gombak	0/11	0/0	1/2	1/13	7.7
Hulu Langat	0/13	10/14	4/15	14/42	33.3
Klang	0/7	5/8	6/10	11/25	44.0
Kuala Lumpur	1/21	0/0	1/3	2/24	9.1
Petaling	3/47	9/13	5/19	17/79	21.5
Putrajaya	2/9	0/0	0/0	2/9	22.2
**Total for Wet Markets**	6/108 (5.6%)	24/35 (68.6%)	17/49 (34.7%)	47/192	24.5
**Abattoir**					
Shah Alam (imported)	5/50	17/40	21/60	43/150	28.6
Banting (local)	8/50	0	0	8/50	1.6
**Total for Abattoirs**	13/100 (13%)	17/40 (42.5%)	21/60 (35%)	51/200	25.5
**Total for Wet Markets + Abattoirs**	19/208	41/75	38/109	98/392	
**Overall Seropositive (%)**	9.1	54.7	34.9		25.0

**Table 4:** Seroprevalence estimates with exact 95% confidence limits (CI), regression univariate and multivariate analysis for *Toxoplasma gondii* antibodies detected in meat samples of cattle, goat and sheep purchased from wet markets in Klang Valley.

	Seropositive Samples (*n*)				Univariate Model		Multivariate Model	
Category	Samples (*n*)	Positive (*n*)	Prevalence (%)	Exact 95% (CI)	Crude OR (95% CI)	*p*-Value	Adjusted OR (95% CI)	*p*-Value
**Meat**								
Cattle	108	6	5.6	2.1,11.7	1.0	-	1.0	-
Goats	35	24	68.6	50.7, 83.1	37.1(12.5,110.3)	<0.001	26.0(12.5,94.6)	<0.001
Sheep	49	17	34.7	21.7,49.6	9.1(3.3-24.8)	<0.001	7.5(2.5,20.8)	<0.001
**Location**								
Gombak	13	1	7.7	0.2,36.0	-	-	-	-
Hulu Langat	42	14	33.3	19.6,49.5	-	-	-	-
Klang	25	11	44.0	24.4, 65.1	-	-	-	-
Kuala Lumpur	24	2	8.3	1.0, 27.0	-	-	-	-
Petaling	79	17	21.5	13.1, 32.2	-	-	-	-
Putrajaya	9	2	22.2	2.8, 60.0	-	-	-	-
**Meat Source**								
Licensed abattoir	118	14	11.9	6.6,19.1	1.0	-	-	0.57
Non-licensed abattoir	74	29	49.2	35.9,62.5	5.9(2.9,12.3)	<0.001	-	-

**Table 5:** Seroprevalence estimates with exact 95% confidence limits (CI), regression univariate and multivariate analysis for *Toxoplasma gondii* antibodies detected in diaphragm samples of cattle, goat and sheep purchased from abattoirs in Selangor.

	Seropositive Samples (*n*)			Univariate Model			Multivariate Model	
Category	Sample (*n*)	Positive (*n*)	Prevalence (%)	Exact 95% (CI)	Crude OR (95% CI)	*p*-Value	Adjusted OR (95% CI)	*p*-Value
**Location**								
Banting	50	8	16.0	7.2–29.1	1.00			
Shah Alam	150	55	36.0	29.0–44.9	2.11	0.080	-	0.372
**Species**								
Cattle	100	19	19.0	11.8–28.1	1.00	-	1.00	-
Goat	40	17	42.5	27.0–59.1	4.9(2.1,11.6)	<0.001	4.2(1.9,10.4)	<0.001
Sheep	60	21	33.3	22.9–45.2	3.6(1.6–7.9)	<0.001	3.1(1.3,6.4)	<0.001
**Sex**								
Male	53	3	5.6	1.2–15.7	1.00	-	-	-
Female	147	48	32.6	25.2–40.9	6.7(2.0–22.7)	0.002	4.4(0.84,18.4)	0.06
